# Elevated remnant cholesterol and the risk of prevalent major depressive disorder: a nationwide population-based study

**DOI:** 10.3389/fpsyt.2024.1495467

**Published:** 2024-11-14

**Authors:** Shiyi Tao, Lintong Yu, Jun Li, Ji Wu, Deshuang Yang, Xuanchun Huang, Tiantian Xue

**Affiliations:** ^1^ Department of Cardiology, Guang’anmen Hospital, China Academy of Chinese Medical Sciences, Beijing, China; ^2^ Graduate School, Beijing University of Chinese Medicine, Beijing, China; ^3^ Department of Integrative Cardiology, China-Japan Friendship Hospital, Beijing, China

**Keywords:** remnant cholesterol, major depressive disorder, dyslipidemia, PHQ-9, NHANES

## Abstract

**Background:**

Remnant cholesterol (RC) has received increasing attention due to its association with a variety of diseases. However, comprehensive population-based studies elucidating the relationship between RC and major depressive disorder (MDD) are limited. The current study aimed to determine the association between RC and MDD in US adults.

**Methods:**

Cross-sectional data of US adults with complete RC and depression information were obtained from the National Health and Nutrition Examination Survey (NHANES) 2005-2018. MDD was evaluated using the Patient Health Questionnaire (PHQ-9). Multivariate logistic regression, sensitivity analysis, and spline smoothing plot method were conducted to explore the relationship between RC and depression. The cut-off point was calculated using recursive partitioning analysis when segmenting effects emerged. The area under the receiver operating characteristic (ROC) curve (AUC), calibration curve, Hosmer-Lemeshow test, the decision curve analysis (DCA), and clinical impact curve (CIC) were employed to evaluate the performance of RC in identifying MDD. Subgroup analyses and interaction tests were performed to explore whether the association was stable in different populations.

**Results:**

A total of 9,173 participants were enrolled and participants in the higher RC quartile tended to have a higher PHQ-9 score and prevalence of MDD. In the fully adjusted model, a positive association between RC and PHQ-9 score and MDD was both observed (β=0.54, 95% *CI* 0.26~0.82; OR=1.43, 95% *CI* 1.15~1.78). Participants in the highest RC quartile had a 0.42-unit higher PHQ-9 score (β=0.42, 95% *CI* 0.15~0.69) and a significantly 32% higher risk of MDD than those in the lowest RC quartile (OR=1.32, 95% *CI* 1.05~1.66). Spline smoothing plot analysis further confirmed the positive and non-linear association between RC and PHQ-9 and MDD. ROC analysis (AUC=0.762), the Hosmer-Lemeshow test (*χ^2^ =* 6.258, *P*=0.618), and calibration curve all indicated a high performance and goodness-of-fit of the multivariate model. DCA and CIC analysis similarly demonstrated a positive overall net benefit and clinical impact for the model. Subgroup analyses and interaction tests suggested that the relationship between RC and depression remained stable across subgroups and was unaffected by other factors other than diabetes, hypertension, or hyperlipidemia.

**Conclusion:**

An elevated RC is associated with a higher risk of prevalent MDD among US adults, especially in those with diabetes, hypertension, or hyperlipidemia. The present results suggested that the management of RC levels and comorbidities may contribute to alleviating the occurrence of MDD.

## 1 Introduction

Mental disorders remained among the top ten leading causes of burden worldwide, with depressive disorders being leading contributors to this burden and without evidence of global reduction in the burden since 1990 (1, 2). Approximately 300 million people globally suffer from depression in 2020, and the number of instances of major depressive disorder (MDD) increased by 53.2 million cases globally following the outbreak of the new coronavirus pneumonia pandemic, resulting in 49.4 million disability-adjusted life-years for MDD (2). Moreover, the World Health Organization (WHO) ranked depression as the third leading cause of global burden of disease in 2004, and it is predicted to overtake it and move to first place by 2030. Antidepressants, pharmacotherapy, lifestyle modifications, psychotherapy, electroconvulsive therapy, and vagal nervous stimulation are all options for treating depression (3–6). Despite the fact that various therapeutic options exist to alleviate the symptoms of depression, none of them are sufficiently capable of resolving the unpleasant impact. Conventional formulations for the treatment of depression have several disadvantages, including limited drug penetration, high dose frequency, adverse reaction, and patient compliance concerns (7, 8). Therapies and lifestyle modifications also need a significant amount of effort to demonstrate their feasibility, which may, in turn, result in passive non-compliance. All of these difficulties hinder the adequate application of available conventional treatments for depression (9). Consequently, an objective and reliable approach for evaluating the onset and progression of depressive disorders is highly desirable but still awaits further development.

Multiple risk factors for depressive disorders exist, including stress, behavioral habits, and socio-demographic factors (1). Furthermore, several studies have identified a possible link between depression and dyslipidemia (10, 11). Remnant cholesterol (RC), also known as triglyceride-rich cholesterol, is mostly composed of very-low-density lipoproteins, intermediate-density lipoproteins, and chylomicron remnants in fasting or non-fasting states, which is defined as total cholesterol (TC) minus high-density lipoprotein cholesterol (HDL-C) minus low-density lipoprotein cholesterol (LDL-C) (12, 13). Initially, RC is gaining popularity due to its tight relationship with cardiovascular diseases. Previous studies have shown that RC is also associated with hypertension, diabetes mellitus and its consequences, nonalcoholic fatty liver disease, and chronic kidney disease. Particularly, the main pharmacological treatment for cardiovascular diseases (CVD) prevention is thought to be reducing LDL-C using statins. Though the guidelines now propose a target LDL-C, there are still hazards associated with CVD, which are referred to as “residual risks”. Besides, the potential connection between cardiovascular diseases and depressive disorders has also been well established (14, 15). To explore whether the RC may also shed light on the complex interaction between cardiovascular disease and depression has great importance. Nevertheless, information on the relationship between RC and depressive disorders is currently limited.

Thus, the present study aimed to determine the relationship between RC and the risk of prevalent depressive disorders using data from the National Health and Nutrition Examination Survey (NHANES) and to assess the performance of RC in identifying MDD.

## 2 Materials and methods

### 2.1 Study population

This study was conducted with data sourced from the National Health and Nutrition Examination Survey (NHANES) carried out from 2005 to 2018. The NHANES database is a national nutrition and health survey of the U.S. population conducted annually since 1999 by the National Center for Health Statistics (NCHS) of the Centers for Disease Control and Prevention (CDC). The survey utilized household questionnaires, telephone interviews, and examinations conducted by medical professionals and trained personnel to collect data and to create a representative sample of the U.S. population through complex multistage, stratified, clustered probability sample. The survey protocol was formally approved by the CDC Institutional Review Board, and all participants gave voluntary informed consent.

**Figure 1:**
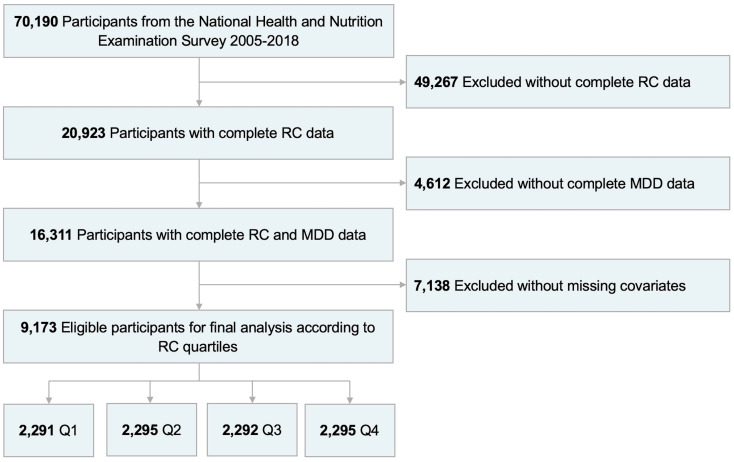
Flowchart of participant selection from NHANES 2005-2018. RC, remnant cholesterol; MDD, major depressive disorder; Q1, quartile 1; Q2, quartile 2; Q3, quartile 3; Q4, quartile 4.

As shown in **Figure 1**, information from NHANES 2005-2018 were used for analysis. Among the 70,190 subjects in the NHANES over the period 2005-2018, a total of 9,173 US adults were enrolled in the present study finally after exclusion of patients with no complete RC data (n=49,267) and MDD information (n=4,612), and with missing covariates (n=7,138).

### 2.2 Major depressive disorder

The Patient Health Questionnaire (PHQ-9) was used to diagnose and assess the severity of depression, a prevalent self-report instrument for screening depression that incorporates the diagnostic criteria for depression as outlined in the Diagnostic and Statistical Manual of Mental Disorders, 4th edition (16). This tool comprises nine items designed to evaluate depressive symptoms experienced within the preceding two weeks. For each symptom question, points ranging from 0 to 3, are associated with the response categories “not at all”, “several days”, “more than half the days”, and “nearly every day”. Individuals with a total PHQ-9 score of 10 or higher were identified as having clinically significant self-reported depressive symptoms (17, 18).

### 2.3 Data collection and definitions

Covariates in the present study included demographic information, medical history, and laboratory data. Demographic data comprised age, gender, race, education level, marital status, family income-to-poverty ratio (FIPR), body mass index (BMI), and waist circumference (WC). Medical history information included PHQ-9 score, cigarette use, diabetes, hypertension, hyperlipidemia, cardiovascular disease (CVD), and chronic kidney disease (CKD). Laboratory data encompassed, white blood cell (WBC), segmented neutrophil (Neu), lymphocyte (Lym), platelet (PLT), hemoglobin (Hb), alanine aminotransferase (ALT), aspartate aminotransferase (AST), alkaline phosphatase (ALP), gamma glutamyl transferase (GGT), serum creatinine (Scr), uric acid (UA), Total cholesterol (TC), triglyceride (TG), LDL-C, HDL-C, fasting plasma glucose (FPG), glycated hemoglobin A1c (HbA1c), iron, phosphorus, potassium, sodium, and calcium. RC was calculated as TC minus HDL-C and LDL-C (13).

The demographic data and medical history information were collected through interviews. Race was categorized into four groups: Mexican American, Non-Hispanic White, Non-Hispanic Black, and other races. Education was stratified into three classes: less than high school, high school or equivalent, and college graduate or above. Marital status was organized into three groups: never married, married or living with partner, and others (widowed, divorced, and separated). Smoking at least 100 cigarettes in entire life was defined as a smoker. Detailed information on the specimen collection, processing, quality assurance, and monitoring are described in the section of biospecimen program in NHANES (https://www.cdc.gov/nchs/nhanes/index.htm).

### 2.4 Statistical analysis

Continuous variables were expressed as mean ± standard deviation (SD) or median [interquartile range (IQR)], and the *t*-test or the Mann-Whitney *U* test was selected for hypothesis testing for those with normal and skewed distributions, respectively. Categorical variables were summarized as percentage-based figures and compared by the Chi-Square test. The t-tests, Mann-Whitney U tests, and Chi-square tests were used for pairwise comparisons across RC quartiles. Multivariate linear and logistic regression model was used to explore the relationship between RC and PHQ-9 score and the risk of prevalent MDD, respectively, and a trend test was performed to test the linear association of RC with depression. RC was treated as a continuous variable in the present analysis, and subjects were grouped based on the RC quartiles for further analysis. Moreover, we established three regression models by adjusting different indicators to control for confounding biases. In addition to the Model 1 without any adjustments for confounders, two other models were fitted. In Model 2, gender, age, race, education level, marital status, and FIPR were modified. Model 3 was a fully adjusted model that took gender, age, race, education level, marital status, FIPR, BMI, WC, smoking, diabetes, hypertension, hyperlipidemia, CVD, and CKD into account. The performance of RC in identifying MDD was assessed using the area under the receiver operating characteristic (ROC) curve (AUC). Meanwhile, we assessed the goodness-of-fit and reliability of the relatively optimal models using calibration curve and Hosmer-Lemeshow test, with *P*>0.05 regarded as an acceptable model. The decision curve analysis (DCA) and clinical impact curve (CIC) were employed to assess the predictive accuracy and clinical value of the model. Subgroup analyses and interaction tests were used to confirm the stability of the association between AIP and PHQ-9 score and MDD across different subgroups.

Statistical analyses were performed using IBM-SPSS (version 26.0, Chicago, IL, USA) and R (version 4.1.2, Vienna, Austria). A two-sided *P*<0.05 was considered statistically significant.

## 3 Results

### 3.1 Baseline characteristics

A total of 9,173 participants with an average age of 60 years were enrolled in this study, of whom 48.63% were male and 51.37% were female. The ranges of RC for quartiles 1-4 were 0.098-0.389, 0.389-0.547, 0.547-0.794, and 0.794-2.072, respectively. The mean PHQ-9 score was 2.00 for all participants and increased with the higher RC quartiles (*P*<0.001). The prevalence of MDD was 9.26% overall, and participants in the higher RC quartile tended to have higher rates of MDD (Q1: 6.98%; Q2: 8.71%; Q3: 9.21%; Q4: 12.11%; *P*<0.001). Among the four RC quartiles, differences with statistical significance were observed in age, gender, race, education level, marital status, FIPR, BMI, WC, smoking, diabetes, hypertension, hyperlipidemia, CVD, CKD, WBC, Neu, Lym, PLT, Hb, ALT, ALP, GGT, Scr, UA, TC, TG, LDL-C, HDL-C, FPG, HbA1c, iron, sodium, and calcium (all *P*<0.05). Compared with the lowest RC group, participants in the increased RC group were significantly more likely to have diabetes, hypertension, hyperlipidemia, CVD, CKD, elevated BMI, WC, WBC, Neu, Lym, PLT, Hb, ALT, ALP, GGT, Scr, UA, TC, TG, LDL-C, FPG, HbA1c, iron and decreased FIPR and HDL-C levels (all *P*<0.05). The difference between quartiles in AST, phosphorus, and potassium did not meet the statistical significance (all *P*>0.05) (**Table 1**).

**Table 1:** Baseline characteristics of study subjects according to RC quartiles.

Variables	Overall	Q1	Q2	Q3	Q4	*P* value
N	9,173	2,291	2,295	2,292	2,295	
PHQ-9 score	2.00 (0.00-4.00)	1.00 (0.00-4.00)	1.00 (0.00-4.00)	2.00 (0.00-4.00)	2.00 (0.00-5.00)	<0.001
MDD						<0.001
Yes	849 (9.26)	160 (6.98)	200 (8.71)	211 (9.21)	278 (12.11)	
No	8324 (90.74)	2131 (93.02)	2095 (91.29)	2081 (90.79)	2017 (87.89)	
Demographics						
Age (years)	60.00 (50.00-70.00)	59.00 (48.00-69.00)	61.00 (50.00-70.00)	61.00 (52.00-71.00)	60.00 (50.00-69.00)	<0.001
Gender						<0.001
Male (n, %)	4461 (48.63)	1042 (45.48)	1120 (48.80)	1097 (47.86)	1202 (52.37)	
Female (n, %)	4712 (51.37)	1249 (54.52)	1175 (51.20)	1195 (52.14)	1093 (47.63)	
Race (n, %)						<0.001
Mexican American	1179 (12.85)	185 (8.08)	272 (11.85)	358 (15.62)	364 (15.86)	
Non-Hispanic White	4332 (47.23)	991 (43.26)	1036 (45.14)	1085 (47.34)	1220 (53.16)	
Non-Hispanic Black	1870 (20.39)	730 (31.86)	544 (23.70)	361 (15.75)	235 (10.24)	
Others	1792 (19.54)	385 (16.80)	443 (19.30)	488 (21.29)	476 (20.74)	
Education levels						<0.001
Less than high school	2265 (24.69)	446 (19.47)	544 (23.70)	642 (28.01)	633 (27.58)	
High school or equivalent	2105 (22.95)	489 (21.34)	538 (23.44)	521 (22.73)	557 (24.27)	
College or above	4803 (52.36)	1356 (59.19)	1213 (52.85)	1129 (49.26)	1105 (48.15)	
Marital status						0.016
Never married	710 (7.74)	208 (9.08)	186 (8.10)	159 (6.94)	157 (6.84)	
Married or living with partner	5804 (63.27)	1426 (62.24)	1415 (61.66)	1497 (65.31)	1466 (63.88)	
Others	2659 (28.99)	657 (28.68)	694 (30.24)	636 (27.75)	672 (29.28)	
FIPR	2.71 (1.33-4.37)	3.09 (1.54-4.62)	2.70 (1.29-4.37)	2.64 (1.35-4.37)	2.44 (1.22-4.37)	<0.001
BMI (kg/m^2^)	28.49 (25.00-33.10)	26.50 (23.18-31.00)	27.98 (24.80-32.27)	29.20 (25.60-33.70)	30.19 (26.80-34.70)	<0.001
WC (cm)	99.80 (91.50-110.30)	94.70 (85.30-104.50)	99.15 (90.70-109.00)	101.30 (93.40-112.23)	104.60 (96.75-114.20)	<0.001
Medical history (n, %)						
Smoking						<0.001
Yes	4453 (48.54)	1013 (44.22)	1087 (47.36)	1105 (48.21)	1248 (54.38)	
No	4720 (51.46)	1278 (55.78)	1208 (52.64)	1187 (51.79)	1047 (45.62)	
Diabetes						<0.001
Yes	1782 (19.43)	302 (13.18)	406 (17.69)	492 (21.47)	582 (25.36)	
No	7391 (80.57)	1989 (86.82)	1889 (82.31)	1800 (78.53)	1713 (74.64)	
Hypertension						<0.001
Yes	4555 (49.66)	971 (42.38)	1123 (48.93)	1188 (51.83)	1273 (55.47)	
No	4618 (50.34)	1320 (57.62)	1172 (51.07)	1104 (48.17)	1022 (44.53)	
Hyperlipidemia						<0.001
Yes	4350 (47.42)	798 (34.83)	1022 (44.53)	1183 (51.61)	1347 (58.69)	
No	4823 (52.58)	1493 (65.17)	1273 (55.47)	1109 (48.39)	948 (41.31)	
CVD						<0.001
Yes	2568 (28.00)	589 (25.71)	622 (27.10)	628 (27.40)	729 (31.76)	
No	6605 (72.00)	1702 (74.29)	1673 (72.90)	1664 (72.60)	1566 (68.24)	
CKD						0.010
Yes	393 (4.28)	84 (3.67)	94 (4.10)	89 (3.88)	126 (5.49)	
No	8780 (95.72)	2207 (96.33)	2201 (95.90)	2203 (96.12)	2169 (94.51)	
Laboratory results						
WBC (1000 cells/uL)	6.40 (5.40-7.80)	5.90 (4.90-7.10)	6.30 (5.20-7.60)	6.60 (5.50-8.00)	6.90 (5.90-8.40)	<0.001
Neu (1000 cells/uL)	3.70 (2.90-4.70)	3.30 (2.60-4.30)	3.60 (2.80-4.60)	3.80 (3.00-4.90)	4.10 (3.20-5.10)	<0.001
Lym (1000 cells/uL)	1.90 (1.50-2.30)	1.70 (1.40-2.10)	1.80 (1.50-2.30)	1.90 (1.60-2.40)	2.00 (1.70-2.50)	<0.001
PLT (1000 cells/uL)	232.00 (195.00-276.00)	223.00 (188.00-266.50)	231.00 (193.00-274.00)	236.50 (200.00-280.00)	238.00 (201.00-284.00)	<0.001
Hb (g/dL)	14.10 (13.10-15.10)	13.80 (12.90-14.70)	14.10 (13.10-15.00)	14.20 (13.20-15.10)	14.50 (13.50-15.50)	<0.001
ALT (U/L)	21.00 (16.00-27.00)	19.00 (15.00-24.00)	20.00 (16.00-26.00)	21.00 (16.00-28.00)	23.00 (18.00-31.00)	<0.001
AST (U/L)	23.00 (19.00-27.00)	22.00 (19.00-27.00)	22.00 (19.00-27.00)	23.00 (19.00-27.00)	23.00 (20.00-28.00)	0.058
ALP (U/L)	69.00 (56.00-84.00)	65.00 (53.00-80.00)	69.00 (56.00-84.50)	70.00 (57.00-85.00)	72.00 (59.00-86.00)	<0.001
GGT (IU/L)	21.00 (15.00-32.00)	18.00 (13.00-26.00)	20.00 (15.00-30.00)	21.00 (16.00-32.00)	26.00 (18.00-41.00)	<0.001
Scr (*μ*mol/L)	76.91 (64.53-90.17)	75.14 (63.65-88.40)	77.79 (64.53-91.05)	76.91 (63.65-90.17)	79.56 (65.42-91.94)	0.029
UA (*μ*mol/L)	327.10 (273.60-386.60)	297.40 (249.80-350.90)	321.20 (267.70-380.70)	333.10 (285.50-392.60)	356.90 (297.40-410.40)	<0.001
TC (mmol/L)	4.99 (4.29-5.72)	4.65 (4.03-5.30)	4.94 (4.24-5.61)	5.04 (4.37-5.74)	5.35 (4.65-6.09)	<0.001
TG (mmol/L)	1.20 (0.86-1.73)	0.68 (0.56-0.77)	1.03 (0.94-1.11)	1.42 (1.30-1.57)	2.23 (1.93-2.73)	<0.001
LDL-C (mmol/L)	2.92 (2.33-3.57)	2.66 (2.15-3.23)	2.95 (2.38-3.57)	3.00 (2.40-3.65)	3.10 (2.43-3.78)	<0.001
HDL-C (mmol/L)	1.34 (1.11-1.66)	1.63 (1.37-1.94)	1.42 (1.19-1.71)	1.32 (1.11-1.55)	1.11 (0.98-1.32)	<0.001
FPG (mmol/L)	5.77 (5.33-6.49)	5.55 (5.16-6.05)	5.72 (5.27-6.27)	5.88 (5.44-6.66)	6.00 (5.50-7.05)	<0.001
HbA1c (%)	5.70 (5.40-6.10)	5.60 (5.30-5.90)	5.60 (5.40-6.00)	5.70 (5.40-6.20)	5.80 (5.50-6.40)	<0.001
Iron (*μ*mol/L)	15.00 (11.50-19.20)	14.50 (11.00-18.60)	15.00 (11.50-19.00)	15.20 (11.60-19.30)	15.60 (12.00-19.50)	<0.001
Phosphorus (mmol/L)	1.16 (1.07-1.29)	1.16 (1.07-1.29)	1.16 (1.07-1.29)	1.16 (1.07-1.26)	1.16 (1.07-1.29)	0.160
Potassium (mmol/L)	4.00 (3.80-4.30)	4.00 (3.80-4.30)	4.00 (3.80-4.28)	4.08 (3.80-4.30)	4.06 (3.80-4.30)	0.067
Sodium (mmol/L)	140.00 (138.00-141.00)	140.00 (138.00-141.00)	140.00 (138.00-141.00)	140.00 (138.00-141.00)	139.00 (138.00-141.00)	<0.001
Calcium (mmol/L)	2.33 (2.27-2.40)	2.33 (2.27-2.38)	2.33 (2.27-2.40)	2.35 (2.27-2.40)	2.35 (2.30-2.40)	<0.001

RC, remnant cholesterol; PHQ-9, patient health questionnaire; MDD, major depressive disorder; FIPR, family income-to-poverty ratio; BMI, body mass index; WC, waist circumference; CVD, cardiovascular disease; CKD, chronic kidney disease; WBC, white blood cell; Neu, segmented neutrophil; Lym, lymphocyte; PLT, platelet; Hb, hemoglobin; ALT, alanine aminotransferase; AST, aspartate aminotransferase; ALP, alkaline phosphatase; GGT, gamma glutamyl transferase; Scr, serum creatinine, UA, uric acid; TC, total cholesterol; TG, triglyceride; LDL-C, low-density lipoprotein cholesterol; HDL-C, high-density lipoprotein cholesterol; FPG, fasting plasma glucose; HbA1c, glycated hemoglobin A1c; Q1, quartile 1; Q2, quartile 2; Q3, quartile 3; Q4, quartile 4.

### 3.2 The relationship between RC and depression

**Table 2**, **3** showed the correlation between RC and depression. The present findings suggested that an elevated RC was associated with a higher PHQ-9 score and an increased risk of prevalent MDD. Moreover, spline smoothing plot analysis further confirmed the positive and non-linear association between RC and PHQ-9 score and MDD (**Figure 2**).

**Table 2:** Cut-off point and segmentation effects of RC on PHQ-9 score.

Items	Outcome
Linear effect	0.54 (0.26, 0.82)
Segmentation effect	
Cut-off point (K)	0.24
< K segment effect	-7.11 (-14.71, 0.50)
> K segment effect	0.60 (0.31, 0.89)
Effect difference	2.75 (2.60, 2.90)
Logarithmic likelihood ratio test	0.048

RC, remnant cholesterol; PHQ-9, patient health questionnaire.

**Table 3:** The relationship between RC and depression.

RC	PHQ-9 score	MDD
	β (95% *CI*)	OR (95% *CI*)
Crude model (Model 1)		
Continuous	1.18 (0.90, 1.46)	1.93 (1.60, 2.33)
Categories		
Q1	0 (reference)	1 (reference)
Q2	0.39 (0.12, 0.65)	1.27 (1.02, 1.58)
Q3	0.41 (0.14, 0.67)	1.35 (1.09, 1.67)
Q4	1.04 (0.77, 1.30)	1.84 (1.50, 2.25)
*P* for trend	<0.001	<0.001
Adjusted model (Model 2)		
Continuous	1.08 (0.80, 1.36)	1.88 (1.54, 2.29)
Categories		
Q1	0 (reference)	1 (reference)
Q2	0.33 (0.07, 0.59)	1.21 (0.97, 1.52)
Q3	0.36 (0.10, 0.63)	1.31 (1.05, 1.63)
Q4	0.92 (0.65, 1.19)	1.74 (1.40, 2.15)
*P* for trend	<0.001	<0.001
Fully adjusted model (Model 3)		
Continuous	0.54 (0.26, 0.82)	1.43 (1.15, 1.78)
Categories		
Q1	0 (reference)	1 (reference)
Q2	0.17 (-0.08, 0.43)	1.12 (0.89, 1.41)
Q3	0.09 (-0.17, 0.35)	1.13 (0.89, 1.43)
Q4	0.42 (0.15, 0.69)	1.32 (1.05, 1.66)
*P* for trend	0.003	0.015

In sensitivity analysis, the RC was converted from a continuous variable to a categorical variable (quartiles). RC, remnant cholesterol; MDD, major depressive disorder; Q1, quartile 1; Q2, quartile 2; Q3, quartile 3; Q4, quartile 4; OR, odds ratio; 95% CI, 95% confidence interval.

Model 1, no covariates were adjusted;

Model 2, Adjusted for gender, age, race, education level, marital status, and FIPR;

Model 3, Adjusted for gender, age, race, education level, marital status, FIPR, BMI, WC, smoking, diabetes, hypertension, hyperlipidemia, CVD, CKD.

**Figure 2:**
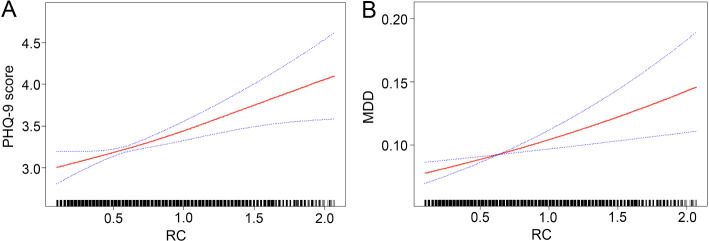
Nonlinear relationship between RC and depression. **(A)**. RC and PHQ-9 score; **(B)**. RC and PHQ-9 score-based MDD symptoms. The red line represents the smooth curve fit between variables, and the blue bands represent the 95% of confidence interval from the fit. RC, remnant cholesterol; MDD, major depressive disorder.

Using recursive partitioning analysis, a statistically significant cut-off point (cut-off point=0.24, *P*=0.048) in the relationship between RC and PHQ-9 score was identified. When the RC was greater than 0.24, it was positively associated with the PHQ-9 score and each unit of increased RC was related to 0.06 increased units of PHQ-9 score. (β=0.60, 95% *CI* 0.31~0.89).

Furthermore, multivariate linear and logistic regression model was conducted to demonstrate the correlation between RC and PHQ-9 score and the risk of prevalent MDD, respectively. As shown in **Table 3**, in the fully adjusted model (Model 3), a positive association between the RC and PHQ-9 score was observed (β=0.54, 95% *CI* 0.06~0.82), showing that each unit of increased RC score was associated with 0.54 increased units of PHQ-9 score. We further converted the RC from a continuous variable to a categorical variable (quartiles) to conduct the sensitivity analysis. Compared with the lowest RC quartile, the PHQ-9 score increased with the higher RC groups. The mean PHQ-9 score of the highest RC quartile was 0.42 units higher than that of the lowest quartile (β=0.42, 95% *CI* 0.15~0.69, *P* for trend=0.003). For MDD, a positive association with statistical significance was also identified between RC and the risk of prevalent MDD. After full adjustment, participants with a unit higher RC had a 43% increased risk of MDD (OR=1.43, 95% *CI* 1.15~1.78). The association remained statistically significant after AIP was treated as quartiles (*P* for trend=0.015), and subjects in the highest RC quartile had a significantly 32% higher risk than those in the lowest RC quartile (OR=1.32, 95% *CI* 1.05~1.66).

### 3.3 Predictive power of RC for MDD

**Figure 3:**
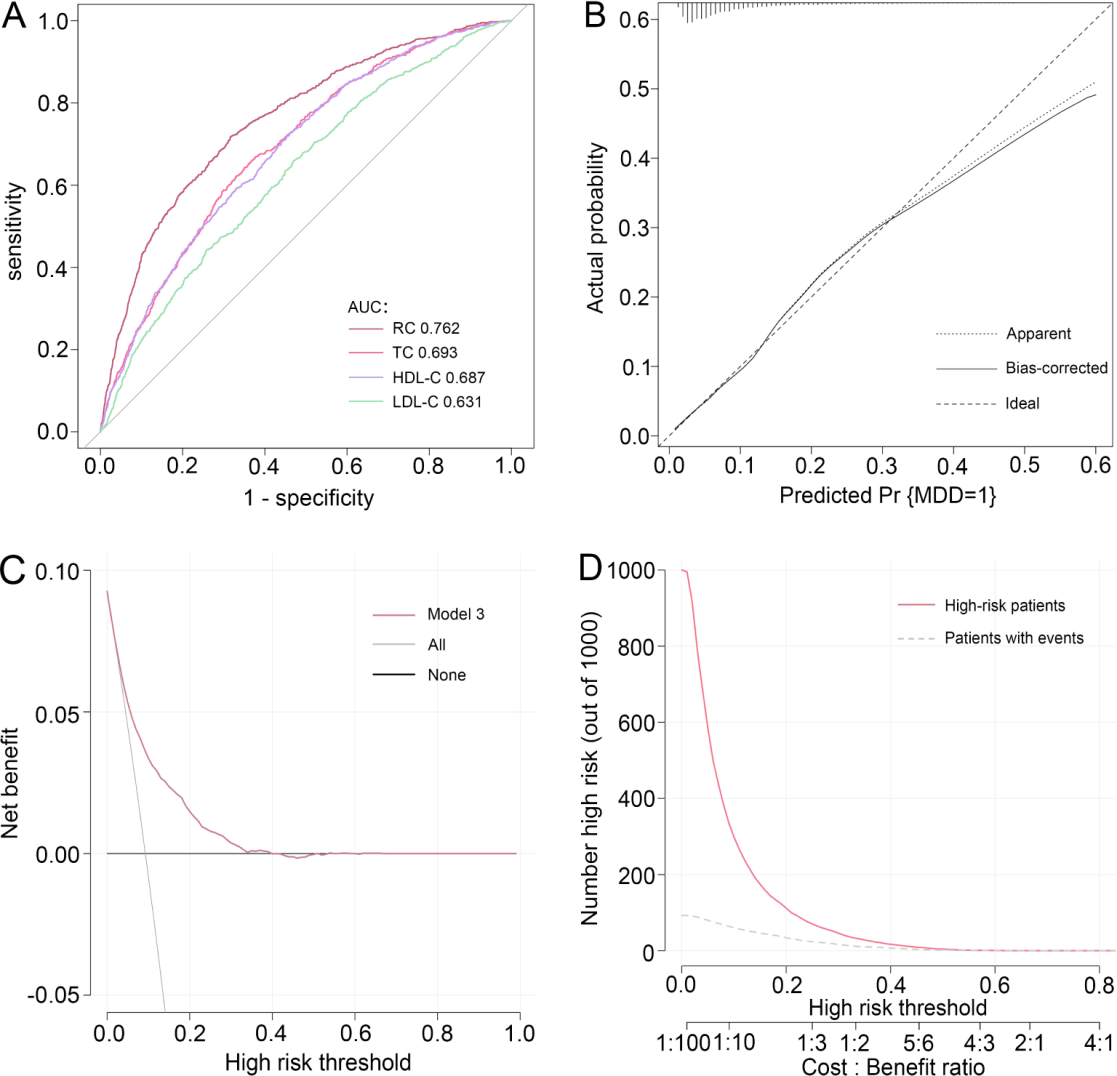
Performance evaluation of RC for predicting MDD. After adjustment for confounding factors, predictive power of RC for MDD was assessed using **(A)** the area under the receiver operating characteristic curve (AUC), **(B)** calibration curve, **(C)** decision curve analysis (DCA), and **(D)** clinical impact curve (CIC) analysis. RC, remnant cholesterol; MDD, major depressive disorder.

To synthetically evaluate the performance of RC in identifying MDD, the ROC curve, calibration curve, Hosmer-Lemeshow test, DCA, and CIC were employed to process the analysis. As shown in **Figure 3**, after adjustment for all potential confounders, ROC analysis showed that the AUC of RC (AUC=0.762) was larger than that of TC (AUC=0.561), HDL-C (AUC=0.687), and LDL-C (AUC=0.631) alone, respectively. The Hosmer-Lemeshow test (*χ^2^ =* 6.258, *P*=0.618) and calibration curve all suggested an excellent performance and goodness-of-fit of the multivariate model. Moreover, DCA and CIC analysis were used to examine the clinical utility of the model, indicating a favorable overall net benefit and clinical impact within most reasonable threshold probability of the model.

### 3.4 Subgroup analysis

**Table 4** showed the results of subgroup analyses and interaction tests stratified by age, gender, BMI, smoking, diabetes, hypertension, hyperlipidemia, CVD, and CKD to evaluate whether the association between RC and MDD was consistent in the general population and to identify any potentially different population settings. Our findings supported that the association between RC and PHQ-9 score and the risk of prevalent MDD was significantly different in the individuals with diabetes, hypertension, or hyperlipidemia (*P* for interaction<0.05), and patients with a unit higher RC had a 97%, 92%, and 82% increased risk of prevalent MDD (OR=1.97, 95% *CI* 1.35~2.87; OR=1.92, 95% *CI* 1.47~2.52; OR=1.82, 95% *CI* 1.38~2.41), respectively.

**Table 4:** Subgroup analysis of the association between RC and depression.

Subgroups	PHQ-9 score		MDD	
	β (95% *CI*)	*P* for interaction	OR (95% *CI*)	*P* for interaction
Age (years)		0.706		0.849
<60	0.58 (0.22, 0.95)		1.41 (1.07, 1.86)	
≥60	0.48 (0.06, 0.90)		1.47 (1.05, 2.05)	
Gender		0.400		0.957
Male	0.45 (0.07, 0.83)		1.44 (1.05, 1.98)	
Female	0.69 (0.27, 1.11)		1.43 (1.06, 1.91)	
BMI (kg/m^2^)		0.078		0.073
<25	-0.00 (-0.65, 0.65)		0.91 (0.51, 1.60)	
≥25	0.65 (0.34, 0.97)		1.58 (1.24, 2.00)	
Smoking		0.750		0.522
Yes	0.50 (0.13, 0.87)		1.36 (1.04, 1.78)	
No	0.59 (0.18, 0.99)		1.56 (1.11, 2.19)	
Diabetes		0.020		0.048
Yes	1.14 (0.56, 1.71)		1.97 (1.35, 2.87)	
No	0.37 (0.06, 0.69)		1.25 (0.96, 1.62)	
Hypertension		0.001		<0.001
Yes	0.96 (0.57, 1.34)		1.92 (1.47, 2.52)	
No	0.02 (-0.39, 0.43)		0.84 (0.57, 1.22)	
Hyperlipidemia		0.008		0.007
Yes	0.85 (0.46, 1.24)		1.82 (1.38, 2.41)	
No	0.09 (-0.31, 0.50)		1.01 (0.71, 1.42)	
CVD		0.342		0.238
Yes	0.73 (0.24, 1.22)		1.27 (0.94, 1.71)	
No	0.45 (0.12, 0.78)		1.63 (1.21, 2.20)	
CKD		0.670		0.645
Yes	0.79 (-0.43, 2.01)		1.21 (0.56, 2.58)	
No	0.52 (0.23, 0.81)		1.45 (1.16, 1.82)	

RC, remnant cholesterol; MDD, major depressive disorder; BMI, body mass index; CVD, cardiovascular disease; CKD, chronic kidney disease; OR, odds ratio; 95% CI, 95% confidence interval.

## 4 Discussion

This nationwide population-based study of 9,173 US adults form NHANES 2005-2018 identified the association between RC and the risk of prevalent depression. The findings implied that an elevated RC level were connected with an increased likelihood of prevalent depression and that RC performs well in predicting MDD. Moreover, this link persisted even after taking into consideration all potential confounding variables, with the highest RC quartile in the population associated with a 32% increased risk. Additionally, associations between RC and depression were significantly different across subgroups stratified by diabetes, hypertension, and hyperlipidemia, showing that elevated RC levels may be related to a higher PHQ-9 score and an increased risk of MDD, particularly in patients with diabetes, hypertension, or hyperlipidemia. Our findings revealed that RC may hold promise for clinical utility in assessing MDD risk and evaluating disease severity.

Depression and stress-related disorders are common mental disorders, affecting approximately one-third of individuals during the life course (19). Recently, increasing evidence has supported that metabolic dysregulation may contribute to the development of psychiatric disorders (20–22). A diverse cohort (23) followed for 30 years revealed that a consistent bidirectional relationship between depressive symptoms and metabolic syndrome was observed, with individuals with more depressive symptoms more likely to develop metabolic syndrome, and metabolic syndrome similarly predicting more depressive symptoms. Most importantly, lipid profiles played a potential role in the risk of developing depressive disorders, and dyslipidemia has been proven to be a risk factor for depression (24). Substantial studies have investigated the relationship between lipid profiles and the risk of prevalent depressive disorders. However, studies examining the association between TC, LDL-C, and HDL-C and the risk of developing depression have yielded inconsistent and even contradictory results (25–29). RC is an important component of cholesterol, accounting for one-third of nonfasting plasma cholesterol and the source of approximately half of the cholesterol present in atherosclerotic plaques (30, 31). Previous studies have shown that RC plays a crucial role in the pathogenesis of various diseases including CVD (32), diabetes (33), liver disease (34), CKD (35), peripheral artery disease (36), and cancer (37). But there is still a lack of research on the relationship between RC and psychiatric disorders, and the predictive power of RC on depression remains to be explored.

Recently, not only has cardiovascular conditions received increased attention, but mental health has also been given more emphasis. Findings of a new study (13) indicated that even when LDL-C was lowered to recommended levels, there was still a substantial residual risk of ischemic heart disease, which may be explained by the association between elevated levels of RC and low-grade inflammation. Furthermore, the relationship of CVD and psychiatric disorders has also been confirmed, with recent data from 21,942 individuals showing that an ideal level of cardiovascular health contributes to the risk reduction of depression (38). Therefore, further investigation of the connection between RC and the risk of prevalent MDD to determine a dependable predictor and to assess the occurrence and development of depressive disorders is of crucial importance. Notably, our study preliminarily offered the population-based evidence that an elevated RC was positively connected with a greater prevalence of MDD and each 1 mmol/L increase in RC corresponded to a 32%. In our subgroup analyses, we found that the relationship between RC and MDD was broadly consistent across a range of demographic and clinical subpopulations, including age, gender, BMI, smoking, CVD, and CKD, suggesting a strong connection that is not significantly affected by these factors. Conversely, individuals with diabetes, hypertension or hyperlipidemia have a higher likelihood of developing depression. However, current studies on the effect of diabetes on the risk of developing depression have produced conflicting results. A nationwide population-based cohort study (39) of about 2 million adults in Korea demonstrated that diabetes severity was positively connected with an elevated risk of developing MDD, and it is feasible to consider screening for depression in those with higher diabetes severity scores. Further studies confirmed that variants of the melatonin receptor 1B gene (MTNR1B) and the prolactin receptor gene (PRLR) were identified as the genetic risk factors for the comorbidity of diabetes and depression (40, 41). Inconsistent with the results, findings from a bidirectional Mendelian randomization study (42) showed a negative correlation between diabetes and the risk of depression onset, indicating that diabetes might reduce the risk of depression. Moreover, hypertension is well known as one of the key risk factors for depression, and the bidirectional and causal relationship between hypertension and depression has also been confirmed in recent years (43). Comorbid hypertension and depressive symptoms were considered to be associated with a higher risk of elevated C-reactive protein levels (44). Experimental study (45) showed that Mfn2-mediated mitophagy activation played a significant role in mitigating depression-like behaviors in hypertension rats. Besides, substantial studies have demonstrated that depression was associated with deteriorating lipid control and higher risk of dyslipidemia (46, 47). Study of 72,235 participants (48) further revealed that the risk of CVD was 1.24-fold higher in dyslipidemia patients with depression than in those without depression.

Several previous studies have suggested the potential mechanism for the close link between elevated RC and the increased risk of depression. RC-induced low-grade inflammation and endothelial dysfunction may be considered as triggers for arterial stiffness (13, 49), and a cross-sectional epidemiological study (50) of 1,510 participants indicated that arterial stiffness of the carotid arteries was found higher in the presence of depression. In addition, due to the lesser size along with high cholesterol content, and increased residence period in blood the remnant lipoproteins, RC is more readily captured and absorbed by macrophages than LDL-C, resulting in faster foam cell formation and inflammatory cytokines secretion (51). Further research found that inflammation may increase the risk of depression through the hypothalamic-pituitary-adrenal axis, and lower levels of inflammatory cytokines may contribute to alleviating depressive symptoms (52, 53). Moreover, the microbiome-lipid metabolic axis has also been regarded as a potential approach to ameliorating depression (54).

There are various strengths to this study. Firstly, NHANES, a population-based sample database that was conducted nationally and adhered to a set process, provided the data utilized in this investigation. Proper NHANES sampling weights were taken into account for all analyses in order to increase the representativeness of the study samples. Furthermore, the represent study computed the cut-off point and identified segmented effects between variables to provide more solid evidence, while concurrently evaluating nonlinear correlations between RC and depressive disorders after controlling for confounding variables. Nevertheless, it is important to note several limitations of the current study. Firstly, we are unable to establish causality between RC and the prevalence of depression due to the cross-sectional study methodology. There is a potential for circular causality and confounding variables in interpreting the link between remnant cholesterol and depressive symptoms. Factors like inflammation, lifestyle, and metabolic conditions might both influence and be influenced by the variables measured, creating complex interdependencies that limit straightforward causal interpretations. Further large-scale prospective studies are still required to confirm our findings. Secondly, information bias can persist even if precautions were made to prevent systematic errors during data collection. Thirdly, although this study provided a timely analysis of RC measurements, the results may not reflect the long-term reality of the included patients. While depression is a more perennial data, data over time in relation to RC could be more useful for this topic.

## 5 Conclusion

The present study demonstrated that elevated RC levels were associated with higher PHQ-9 scores and a greater prevalence of prevalent MDD. Our findings highlight the importance of the management of RC even in the presence of normal lipid testing in identifying patients at risk of MDD. Furthermore, managing comorbidities such as diabetes, hypertension, and hyperlipidemia may assist to minimize the risk of developing MDD. Nevertheless, further large-scale prospective studies are still required to provide more robust evidence to validate our findings.

## Data Availability

The original contributions presented in the study are included in the article/supplementary material. Further inquiries can be directed to the corresponding author.
